# Epigenetic reprogramming in mouse and human primordial germ cells

**DOI:** 10.1038/s12276-024-01359-z

**Published:** 2024-12-13

**Authors:** Sun-Min Lee, M. Azim Surani

**Affiliations:** 1 Department of Physics, Konkuk University, Seoul, Korea; 2 Wellcome Trust/Cancer Research UK Gurdon Institute, Henry Wellcome Building of Cancer and Developmental Biology, University of Cambridge, Cambridge, UK; 3 Physiology, Development and Neuroscience Department, University of Cambridge, Cambridge, UK

**Keywords:** Germline development, Epigenetics

## Abstract

Primordial germ cells (PGCs) are the precursors of sperm and eggs. They undergo genome-wide epigenetic reprogramming to erase epigenetic memory and reset the genomic potential for totipotency. Global DNA methylation erasure is a crucial part of epigenetic resetting when DNA methylation levels decrease across the genome to <5%. However, certain localized regions exhibit slower demethylation or resistance to reprogramming. Since DNA methylation plays a crucial role in transcriptional regulation, this depletion in PGCs requires mechanisms independent of DNA methylation to regulate transcriptional control during PGC reprogramming. Histone modifications are predicted to compensate for the loss of DNA methylation in gene regulation. Different histone modifications exhibit distinct patterns in PGCs undergoing epigenetic programming at the genomic level during PGC development in conjunction with changes in DNA methylation. Together, they contribute to PGC-specific genomic regulation. Recent findings related to these processes provide a comprehensive overview of germline epigenetic reprogramming and its importance in mouse and human PGC development. Additionally, we evaluated the extent to which in vitro culture techniques have replicated the development processes of human PGCs.

## A Unique Journey of Germ Cells with Dramatic Epigenetic Changes

Primordial germ cells (PGCs), which eventually become eggs or sperm, undergo unique and important changes early in their development that are crucial for forming a complete organism after fertilization. Recent studies have advanced our understanding of these changes. This review explains how certain processes, such as adding chemical markers to DNA (DNA methylation) and modifying proteins around DNA (histone modifications), control the development of PGCs in humans and mice. It also explores the replication of these processes in the lab using human stem cells. This review provides important insights into the impact of these changes on reproduction and offers potential new avenues for treating infertility.

This summary was initially drafted using artificial intelligence, then revised and fact-checked by the author.

## Introduction

Germ cells generate a totipotent zygote at fertilization. In humans, primordial germ cells (PGCs), the precursors to eggs and sperm, emerge around the 2nd week of embryonic development^1,2^. Between Weeks 3 and 5, these PGCs migrate to the gonads, where they undergo sex-specific differentiation^3,4^. Following prolonged development and quiescence, they mature into functional sperm and eggs at puberty. In other mammalian species, including mice, similar pathways of germ cell development occur. However, differences exist depending on the species, including unique regulatory networks and kinetics^5–7^.

During the development of PGCs, there are significant epigenetic reprogramming processes, including the erasure of global DNA methylation. Two rounds of reprogramming take place during early development^8^. In the first round, the distinct epigenetic and structural traits of the paternal and maternal genomes are erased through a reset mechanism. Global DNA methylation levels continue to decrease until the blastocyst stage, when the inner cell mass (ICM) is first formed. As the embryo implants during gastrulation, there is a concurrent global remethylation of the genome, which is thought to play a role in lineage restriction. The process in PGCs differs from that in preimplantation embryos. In PGCs, demethylation, including X chromosome reactivation, is almost complete. The developing embryos retain methylation in the imprinted regions, whereas these modifications are comprehensively erased in PGCs^9^.

In addition to genome-wide changes in DNA methylation, global and dynamic changes in histone modifications occur during differentiation processes^9^. The dynamics of histone modifications during human PGC (hPGC) differentiation were initially observed by immunostaining and comparing the fluorescence intensity between PGCs and the surrounding somatic cells. These analyses revealed that the trimethylation of histone H3 at lysine 27 (H3K27me3), a repressive histone modification, produced a stronger signal in migratory-stage PGCs than in the surrounding somatic cells. However, this signal became depleted by Weeks 7–9. Another repressive mark, H3K9me2, was present at lower levels in PGCs than in somatic cells, whereas H3K9me3 remained at similar levels in both PGCs and somatic cells. These changes in histone modifications, occurring alongside extensive DNA demethylation, are hypothesized to play a role in transcriptional regulation and chromatin structure alterations during PGC differentiation.

The development of advanced techniques has contributed to more recent breakthroughs in understanding the mechanism of PGC development. Comprehensive analyses of the DNA methylome and histone modifications during in vivo PGC development in mice and humans have provided a better understanding of the sequential reprogramming processes. Additionally, research aimed at replicating these differentiation processes in vitro has progressed, leading to the differentiation of human PGC-like cells (hPGCLCs) accompanied by epigenetic reprogramming. This review considers the latest advances in the field of human germline biology.

## Germ cell development in mice and humans

Investigations into mammalian germ-cell development have focused predominantly on mouse models. Embryo development commences with fertilization with the formation of a zygote. Subsequent cleavage divisions lead to the development of a morula, which then matures into a blastocyst—a hollow structure comprising an outer trophoblast layer and an ICM. Following implantation, the embryo undergoes gastrulation, marking the onset specification of mouse PGCs (mPGCs)^10^. mPGCs are specified at embryonic day (E) 6.25 in the posterior epiblast and then migrate into the hindgut before colonizing the genital ridge by E10.5–11.5^11,12^. At E13.5, XY and XX mPGCs initiate sex-specific germline programming in response to gonadal cues^13,14^.

In humans, PGCs emerge around Week 2 in gastrulating embryos from the epiblast at the posterior end shortly after implantation. Subsequently, hPGCs migrate from the yolk sac wall through the hindgut by Weeks 5–6, eventually colonizing the developing genital ridge. The germ cells in human embryos prior to 11 weeks postfertilization are called PGCs. After 11 weeks postfertilization, these cells in male and female embryos are termed gonocytes (pro-spermatogonia) and oogonia, respectively. Single-cell RNA-seq analysis of developing human gonads between Weeks 4 and 26 postfertilization revealed heterogeneity and the developmental pathways of human germ cells^15^. hPGCs in both male and female gonads (gonadal hPGCs) underwent mitotic division, with ~50% of the germ cells showing active proliferation. Male hPGCs displayed heterogeneous entry into the mitotic arrest phase starting at Week 9, whereas female hPGCs commenced meiosis followed by oogenesis around Week 11. Similarly, in mice, PGCs in male gonads entered mitotic arrest at ~E13.5, whereas those in female gonads entered meiotic prophase^13,14^.

Sequential expression changes in PGCs occur dynamically in line with their developmental progression^15^. The expression of early germ cell genes, such as *PRDM1*, *PRDM14*, and *TFAP2C*, continues from the start of hPGC specification, while other genes, such as *DMRT1*, *CDH5*, and *DAZL*, are detected during the migratory phase^16,17^. Following the migratory phase, once the hPGCs have colonized the gonads, increases in the expression of *DDX4* and *PIWIL2* are observed. Notably, the expression of genes that regulate the mitotic arrest of hPGCs in males or prepare for meiosis in females exhibits differential gene expression depending on the sex of the developing fetus^15^. Although comparable stage-specific regulatory mechanisms are observed in mice, the factors and mechanisms involved differ to some extent.

The development of PGCs follows a similar path in both mice and humans, but notable mechanistic distinctions exist between the two species. In mice, Prdm1, Prdm14, and Tfap2c^18,19^ are the critical transcription factors for PGC specification and development, whereas in humans, SOX17 and PRDM1 are crucial for PGC specification^20^. TFAP2C is a key factor during hPGC specification, with SOX17-activating enhancers employed to establish the core germline program^21^. Additionally, SOX17 and TFAP2C collaborate with GATA3 or GATA2, which are immediate BMP effectors, to induce hPGCLCs^22^. Importantly, SOX17 is not a regulator of mPGCs, whereas *SOX2* expression in mPGCs is strongly repressed in hPGCs^9,20,23^. The divergence of the molecular mechanism is correlated with the differences in the embryonic structures of mouse and human postimplantation embryos; mouse embryos develop as egg cylinders, whereas human embryos develop as bilaminar discs^24,25^. Differences are also observed in the tempo of mouse and human germ cells^2,26^. Recent research findings have shown that mouse and human PGCs exhibit both commonalities and differences in the regulation of epigenetic reprogramming.

## DNA methylation and demethylation

The DNA methyltransferases DNMT3A and DNMT3B are responsible for de novo DNA methylation, whereas DNMT1 acts as a maintenance methyltransferase, primarily targeting hemi-methylated CpG sites. In mammals, DNA methylation occurs predominantly at CpG dinucleotides, resulting in 5-methylcytosine (5mC)^27^. Cytosine demethylation occurs through active and passive mechanisms^28^. Passive demethylation is the gradual loss of methyl groups that are not restored during DNA replication. Active demethylation involves ten-eleven translocation enzymes (TET1, TET2, TET3) that oxidize 5mC to 5-hydroxymethylcytosine (5hmC), 5-formylcytosine (5fC), and 5-carboxycytosine (5caC), which are often coupled with thymine-DNA glycosylase (TDG)-mediated base excision repair^29,30^.

Global demethylation in preimplantation embryos and PGCs involves both passive and active processes^9,31–33^. Passive demethylation results from the repression of DNMTs and UHRF1, which normally direct DNMT1 to replication foci^9,33,34^. In contrast, active demethylation is associated with increased expression of the TET family in preimplantation embryos and PGCs. In human blastocysts, *DNMT1* and *UHRF1* are repressed, whereas *TET1* and *TET2* are highly elevated^35^. Similarly, hPGCs repressed *UHRF1*, *DNMT3A*, and *DNMT3B*, whereas *TET1* and *TET2* were enriched in hPGCs compared with those in somatic cells^9^.

The bisulfite sequencing method is commonly used to measure DNA methylation rates at single-base resolution. Sodium bisulfite rapidly deaminates unmodified cytosine to uracil, which is then converted to thymine (T) after polymerase chain reaction. Moreover, 5mC and 5hmC remain indistinguishable as C^36^. Recent findings measuring 5hmC separately revealed levels below 10% in early mouse embryos and PGCs^37^. However, it is crucial to recognize that 5hmC also exhibits its own dynamics at the genomic level.

## Genome-wide DNA methylation reprogramming in PGCs

### DNA methylation dynamics in mouse PGC development

**Fig. 1:**
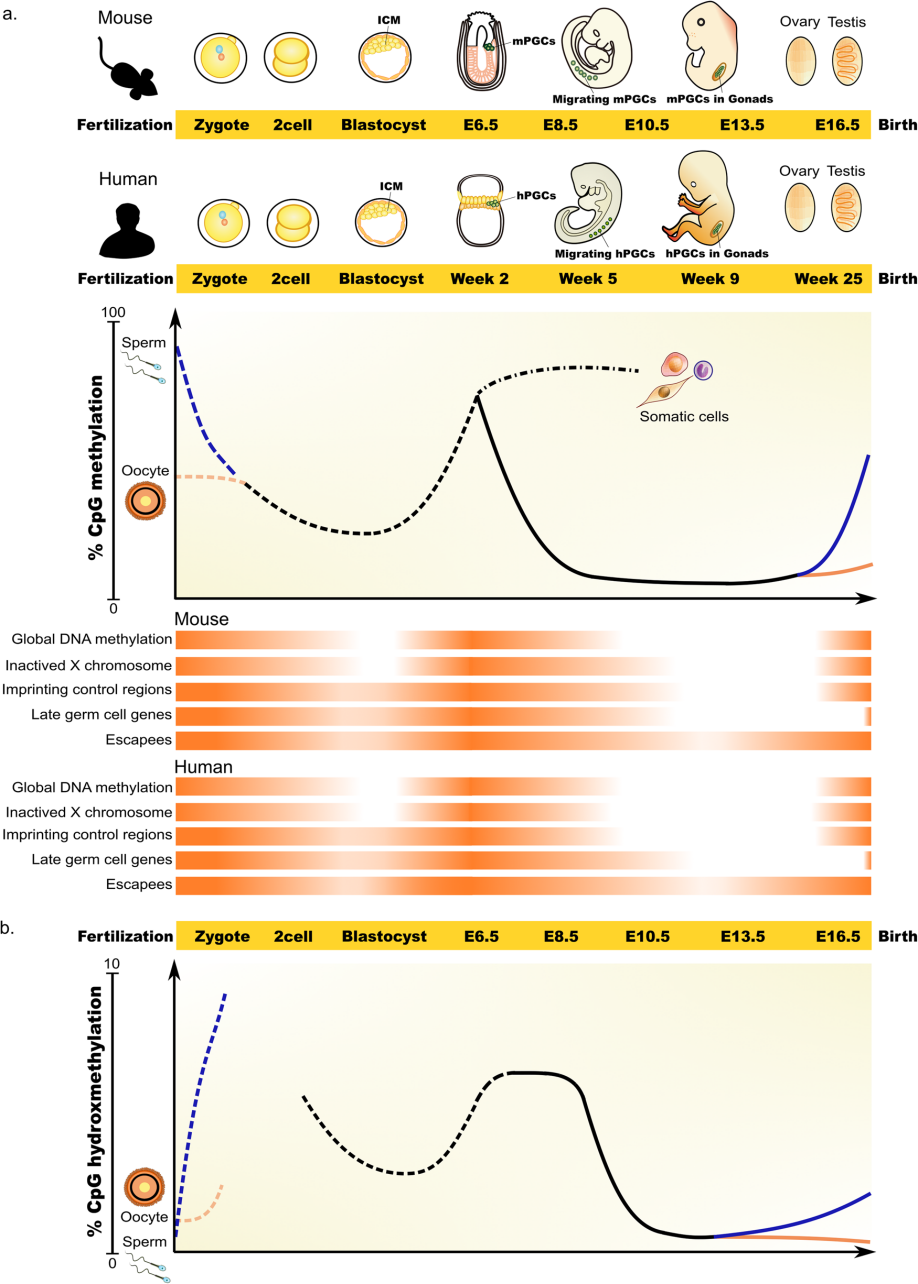
DNA methylation reprogramming in early embryos and the germlines of mice and humans. **a** Schematic showing the level of DNA methylation in the mouse and human genomes. Genome-wide demethylation occurs asymmetrically in the paternal and maternal genomes after fertilization, resetting the epigenome for naïve pluripotency at the blastocyst stage (dotted line). Methylation re-establishment occurs around the time of implantation. Unlike somatic cells (dash-dotted line), PGCs (solid line) undergo global demethylation during the onset of migration and early settlement at the genital ridge. While methylation is reestablished in prenatal male germ cells, in oocytes, this process is not completed until puberty. DNA methylation levels during early embryo and germline development are represented by the orange color density for the indicated regions. **b** Schematic showing the level of DNA hydroxymethylation (5hmC) in the mouse genome during development. The dotted line represents the 5hmC levels in the early embryo after fertilization. The solid line indicates the 5hmC levels in the germline.

After fertilization, the highly methylated epigenome of sperm, and to a lesser extent that of oocytes, is globally erased during progression to the ICM (Fig. 1a). The median methylation level of 80% in sperm rapidly decreases to 38% in the early male pronucleus (PN), 32% in the oocyte, and 28% in the early female PN^38,39^. It finally decreases to ~20% in the ICM of the E3.5 embryo^40^. De novo methylation occurs after E4.5, with global levels recovering to ~75% in the E6.5 epiblast^41–43^. Thus, immediately after their specification from the epiblast, mPGCs likely have similar levels of DNA methylation as in the epiblast. However, during their migration, mPGCs undergo dramatic DNA demethylation, which is not observed in neighboring somatic tissues.

At E9.5, mPGCs migrate through the hindgut endoderm when the methylation level significantly decreases to 30%^41^. From E9.5, methylation levels gradually decrease to ~5% in E11.5 mPGCs, followed by a further decrease to ~4% in E13.5 mPGCs^43^. Between E13.5 and E16.5, female mPGCs maintain low levels of methylation (<10%), corresponding to the stage at which they enter meiosis and then undergo arrest. In comparison, E16.5 male mPGCs exhibit an increase in methylation to ~60%, indicating robust de novo methylation initiation in male mPGCs before birth^43,44^. The surrounding somatic cells maintained higher methylation levels at ~65% (Fig. 1a).

Global erasure of DNA methylation occurs in both genic and intergenic regions, including CpG islands (CGIs), during mPGC development. However, specific genomic regions exhibit relatively delayed loss of DNA methylation (Fig. 1a)^41,44^. For example, CGIs on the X chromosome in female mPGCs undergo protracted DNA demethylation throughout mPGC differentiation, with significant methylation removal between E10.5 and E14.5. Additionally, a group of promoter CGIs retain DNA methylation to some extent at E11.5 and undergo demethylation thereafter. These CGIs are associated with ‘late’ germ cell genes involved in meiosis and gamete generation. Most known imprinting control regions (ICRs) are mostly demethylated in E13.5 mPGCs, but some maternally imprinted ICRs retain partial methylation. At E16.5, all three known paternally imprinted ICRs showed increased methylation levels, but this was not observed in female mPGCs, indicating a pattern of sex-specific differences in DNA methylation.

### DNA methylation dynamics in human PGC development

In humans, the median DNA methylation level of the paternal genome decreases from 82% in sperm to 53% in early male PN. In comparison, the median DNA methylation level of the maternal genome decreases from 55% in the mature oocyte to 51% in the early female PN. During the subsequent development of blastocysts, the methylation level in the ICM decreases to ~30–35%^9,45,46^ (Fig. 1a), which is also the case in the trophectoderm^45,47^.

Following implantation, DNA methylation in the human embryo is restored to over 70%. Genome remethylation occurs much faster in epiblast cells than in cells of the primitive endoderm (PE) and trophectoderm (TE) lineages^48^. In cultured human peri-implantation embryos, the average methylation level of the epiblast is >60%, whereas the average methylation levels of the PE and TE are approximately 40% and 50%, respectively. Following their specification and migration, hPGCs experience a decrease in DNA methylation levels, unlike somatic cells. Whole-genome analysis of the DNA methylome of hPGCs from Weeks 5.5 to 9 revealed that, at Week 5.5, hPGCs became globally hypomethylated, with a median CpG methylation of 16%. This percentage decreased to a basal level of 4.5% by Week 7 and remained low at Week 9 in both female and male hPGCs (Fig. 1a)^9^. A gradual and slight increase in DNA methylation was observed in testicular germ cells from Weeks 17 to 24. Conversely, in ovarian germ cells, the DNA methylation level of female germ cells remained constant between Weeks 10 and 21^49^. Single-cell analysis of the DNA methylome revealed no significant differences in DNA methylation levels among various phases of gonadal PGCs, including comparisons between the mitotic and mitotic-arrest phases in males, as well as among the mitotic phase, retinoid acid (RA) signaling-responsive, meiotic prophase, and oogenetic phase in females^49^.

hPGCs undergo a comprehensive wave of epigenome resetting across all genomic features. However, some genes associated with the piRNA metabolic process, sexual reproduction, and germ cell development (late germ cell genes), including Kruppel-associated zinc finger genes, exhibit gradual promoter demethylation between Weeks 5.5 and 9 of hPGC development (Fig. 1a)^9^. This pattern is similar to that observed in mPGCs. Conversely, the demethylation of ICRs and CGIs on the X-inactivated allele occurs more rapidly in hPGCs than in mice. The inactivated X chromosomes in hPGCs are already transcriptionally reactivated in 4-week-old embryos, and hypomethylation of CGIs on the X chromosome is observed in Week 5.5 hPGCs. Approximately 27% of the ICRs tested retained methylation at Week 5.5, and by Week 10, they were free of methylation^9,50^.

## Escapees in human PGCs as potential candidates for intergenerational epigenetic inheritance

During the global reprogramming process, DNA methylation levels decrease to <5% across the genome. However, some regions retain partial DNA methylation, making them potential candidates for the transmission of parental epigenetic information to the next generation (Fig. 1a)^9,41,51^. These regions are referred to as escapees, and in both mice and humans, over 90% of these regions are associated with transposable elements (TEs). The remaining repeat-poor escapees in humans are associated with genes that are often linked to metabolic and neurological disorders^9^. Additionally, certain single-copy loci in both mice and humans have been shown to retain DNA methylation^9,41^.

TEs vary in their evolutionary age depending on the time of their insertion into the genome. The types and combinations of TEs present also differ between species^52^. In humans, the major groups of TEs include long and short interspersed elements (LINEs and SINEs, respectively), long terminal repeats (LTRs), and variable number of tandem repeats-Alu elements (SVAs). Among the subfamilies of LINEs, the evolutionarily younger L1 subfamily exhibits higher residual methylation levels than the older L2 subfamily. Similarly, the younger Alu subfamily of SINEs has higher levels of residual methylation than the older MIR subfamily^50^. SVA loci, which are hominid-specific TEs, also remain partially methylated. Notably, half of the SVA loci maintain more than 30% methylation across all stages of hPGC development^9^. In mice, intracisternal A particles (IAPs) retain substantial methylation in mPGCs from E9.5 to E16.5. The maintenance of DNA methylation in PGCs may serve as a minimal defense mechanism against retrotransposition, given that the L1, Alu, SVA, and IAP subfamilies harbor active members capable of retrotransposition^53,54^. Escapees from DNA methylation have also been identified in pericentromeric satellite repeats and subtelomeric regions, suggesting a potential role in maintaining chromosome stability. However, the effects of these escape events on developmental processes and their transmission to subsequent generations remain unclear.

## DNA hydroxymethylation in mouse and human PGCs

Another methylated derivative, 5hmC, also undergoes dynamic changes and is involved in both global demethylation processes and genome regulation in PGCs^9,51^. In hPGCs at Week 4, reduced global levels of 5mC compared with those in the surrounding soma were observed, and this lack of 5mC persisted in gonadal hPGCs from Weeks 7 to 9 as observed by immunostaining. However, 5hmC was evident in the hPGC at Week 4, appearing as bright foci in certain nuclear regions, but its presence gradually diminished by Week 9^9^.

Recent technological advancements have facilitated the discrimination and base-resolution observations of both 5mC and 5hmC^27^. Consequently, research on the dynamics of these modifications during mouse PGC and early embryo development has emerged (Fig. 1b). After fertilization, there was a notable increase in 5hmC levels in the male PN, reaching an average of 9% (up from 0.7% in sperm). In comparison, the female PN exhibited a more modest increase to 2% (up from 1.6% in oocytes)^37^. The overall 5hmC level subsequently gradually decreased after the two-cell stage, with a subsequent increase observed in postimplantation epiblasts. Comparable levels of 5hmC, ~4.5%, were detected between E6.5 epiblasts and E9.5 mPGCs. However, a sharp decrease in global 5hmC levels was observed in E11.5 mPGCs (~2%), which decreased further in E13.5 mPGCs (<1%). During de novo DNA methylation in E16.5 male germ cells, a marginal elevation in 5hmC levels (~2%) was noted, in contrast with the absence of such an increase in female germ cells.

The erasure of DNA methylation at the genome-wide level primarily occurs through passive demethylation during replication rather than active demethylation mediated by TET enzymes^32,51^. However, for specific regions crucial for PGC differentiation and requiring temporal regulation, such as imprinting regions and genes involved in gamete generation, active demethylation plays a significant regulatory role. For example, the promoters of *Dazl*, *Ddx4*, and some meiotic genes are enriched with 5hmC signals and are not transcriptionally activated in *Tet1*−/− mPGCs^32^. The specific genomic regulatory functions and significance of 5hmC in PGCs are gradually being elucidated.

## The transcriptome landscapes of mouse and human PGCs undergoing global demethylation

In somatic cells, DNA methylation at promoters is often linked to lower gene expression, whereas methylation within gene bodies is associated with higher gene expression^55,56^. These findings indicate a close association between DNA methylation changes and transcriptional regulation. Although there is widespread erasure of DNA methylation in mouse and hPGCs, there is no fundamental shift, with similar numbers of genes exhibiting high, intermediate, and low transcription levels in all cell types, including PGCs^9,41^. Excluding specific genes, such as those involved in late germ cell development, the global loss of methylation at promoters does not result in a significant shift in transcriptional regulation. However, this does not mean that the role of DNA methylation in gene expression regulation is wholly abolished in PGCs. Although less pronounced than that in somatic cells, a negative correlation between promoter methylation and gene expression has been observed in hPGCs. Additionally, methylation in gene body regions is positively correlated with expression levels in hPGCs^50^.

DNA methylation plays a significant role in regulating the expression of TEs^57^. However, in PGCs, DNA demethylation of TEs is not accompanied by their global derepression, which is consistent with the overall transcription of other genes^9,41^. For example, no significant derepression of the L1 and Alu subfamilies was observed in hPGCs, where DNA methylation was lower than that in somatic cells. However, SVA elements were significantly negatively correlated with methylation and expression in hPGCs. As DNA methylation decreased from Weeks 5 to 9, the expression of these genes increased progressively^9^.

## Histone modifications accompanying reprogramming in PGCs

### Histone modification dynamics in mouse PGC development

During the differentiation process of PGCs, histone modifications, along with DNA methylation, were observed to undergo changes at the genomic level. The distribution of H3K27me3 and H3K9me3 in mPGCs has been analyzed^58^. A substantial reduction in the number of H3K27me3 peaks was observed in both male and female mPGCs from E10.5 to E13.5. The extent of this reduction varied between sexes, with male mPGCs retaining ~20% of the H3K27me3 peaks compared with E10.5, whereas female mPGCs retained ~36%, indicating a more pronounced decrease in H3K27me3 in males. In contrast, the number of H3K9me3 peaks significantly increased in males, with a threefold increase from E10.5 to E13.5, whereas females maintained a similar number of peaks at both stages. However, Western blot analysis of H3K27me3 and H3K9me3 levels in E13.5 mPGCs from male and female mice did not reveal any differences in the overall levels. These findings suggested that the observed differences in peak numbers might be due to variations in the targeting of histone-modifying complexes^58^.

An examination of the changes in H3K27me3 and H3K9me3 in promoter regions following DNA demethylation revealed that H3K9me3 levels generally decreased at most promoters. In contrast, H3K27me3 levels did not change overall but increased at the promoters of certain genes^58^. An increase in H3K27me3 from E10.5 to E13.5, which coincides with a decrease in DNA methylation or the maintenance of high levels of H3K27me3 despite a reduction in DNA methylation, was observed in promoters characterized by high CpG density. Additionally, the promoters of germline-specific genes, such as *Stra8*, *Sycp1*, and *Dnmt3l*, were enriched with H3K27me3. The deletion of *Ezh2*, the main enzyme responsible for this histone modification, resulted in the derepression of these genes, indicating their transcriptional regulation by H3K27me3 following DNA demethylation. The deletion of *Ezh2* had a stronger effect on female mPGCs than on male mPGCs, including the upregulation of genes involved in meiosis.

H3K27me3 also plays a role in TE regulation in PGCs. Compared with somatic cells, mPGCs (at E11.5 and E13.5) demonstrated stronger enrichment of H3K27me3 at specific TE loci, such as SINE-B2, SINE-B4, LINE-L1, and LTR-ERVK^59^. Compared with those at E11.5, the TEs that presented an increase in H3K27me3 levels were mainly those that had lost transposition activity, such as LTR29 and LTR59. In contrast, the TEs that presented increased H3K9me3 alone were evolutionarily young and transcriptionally competent. H3K9me3 appears to play a more prominent role in TE regulation, as over 90% of H3K9me3 peaks at E13.5 are located within 1 kilobase of TEs^58^.

### Histone modification dynamics in human PGC development

**Fig. 2:**
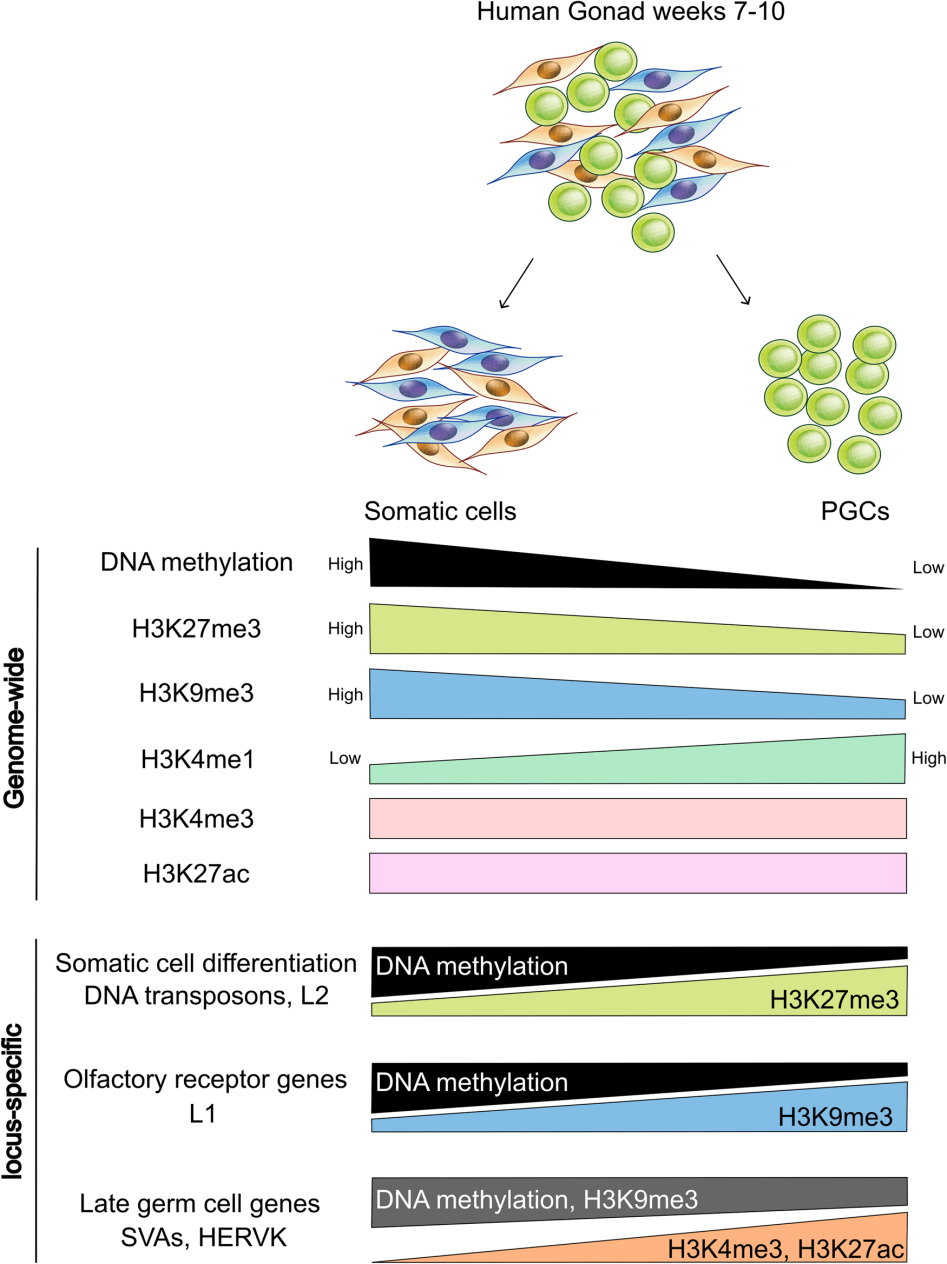
Global and locus-specific epigenetic distinctions between human PGCs and surrounding somatic cells. Sequencing results from human PGCs and surrounding somatic cells revealed genome-wide differences in DNA methylation and histone modifications, as well as locus-specific variations. Relative levels of DNA methylation and histone modifications between somatic cells and PGCs are depicted for olfactory receptor genes (ORGs), late germ cell genes, genes involved in somatic cell differentiation, and various subtypes of transposable elements (TEs).

Compared with somatic cells, hPGCs exhibit different patterns of histone modifications^60^. The prominent feature observed in hPGCs (Weeks 7–10) is the markedly lower occupancy of the repressive histone modifications H3K27me3, H3K9me3, and H2aK119ub than in somatic cells. Conversely, there is a greater occupancy of H3K4me1 in hPGCs. No significant genome-wide alterations were detected when the levels of active histone modifications (H3K4me3 and H3K27ac) and chromatin accessibility, as indicated by ATAC-seq signals, were compared between hPGCs and somatic cells (Fig. 2). A comparison of H3K27me3 levels at Weeks 7 and 9 in hPGCs revealed a more significant decrease in H3K27me3 levels in females than in males at Week 9. This result differs from findings in mouse PGCs. Human X chromosome reactivation was completed before Week 7. The X chromosome–encoded H3K27me3 demethylase *KDM6A* was expressed at higher levels in female hPGCs than in male hPGCs at Weeks 7–8^60^. Conversely, X chromosome reactivation was still ongoing between E10.5 and E14.5 in mice. Additionally, Western blot analysis revealed higher global H3K27me3 levels in mPGCs than in somatic cells at E12.5 but not in hPGCs at Weeks 8–9, which corresponds to the same stage^60^. Human and mouse PGCs displayed different kinetics and sex-specific regional regulation of H3K27me3 during PGC differentiation.

Over 60% of promoters marked by DNA methylation in somatic cells did not show enrichment of active or repressive histone modifications that are observed in hPGCs^60^. Compensation for reduced DNA methylation levels in hPGCs by H3K9me3 or H3K27me3 affected only a small percentage of promoters. The occurrence of increases in active histone marks, chromatin accessibility, or transcriptional activity was also rare. However, reduced promoter methylation was linked with H3K4me1 acquisition^60^.

Some promoters with both H3K9me3 and DNA methylation marks in somatic cells either maintained H3K9me3 after the loss of DNA methylation in hPGCs or presented increased H3K9me3 in hPGCs upon DNA demethylation. These genes were associated mainly with sensory perception^60^. Notably, olfactory receptor genes (ORGs) were clustered (Fig. 2). ORGs have evolved according to the environmental conditions faced by different species, and it is estimated that mice have ~1200 functional ORGs, whereas the corresponding number for humans is only ~350^61^. This outcome is attributed to genetic alteration processes, including copy number variants (CNVs), with ORGs in humans exhibiting a high level of CNVs^62^. Homozygous deletions and amplifications have revealed a significant negative correlation with DNA methylation^63,64^. hPGCs might maintain a repressive chromatin status to prevent unwanted structural changes in ORGs during germline development.

Genes that exhibited an increase in the active mark H3K4me3 after DNA demethylation in hPGCs were mainly involved in gamete generation, such as the genes *DAZL*, *DDX4*, and *MAEL*, or encoded KRAB-zinc finger transcriptional regulators^60^. These genes also exhibited the H3K9me3 mark, thus retaining bivalent modifications (Fig. 2). In Week 9, there was a notable increase in the number of genes with male-specific promoters enriched with H3K27me3, which was not the case in Week 7 hPGCs. In male hPGCs, there was greater enrichment of H2aK119ub than in female hPGCs. H2aK119ub is mediated by the PRC1 complex, which contains CBX chromodomain proteins that bind to H3K27me3^65^. The distribution of H3K27me3 can influence the distribution of H2aK119ub. Most promoters that lost H3K27me3 and gained H3K4me3 in female hPGCs between Weeks 7 and 9 presented moderate transcriptional induction. Genes in this category were associated with cell adhesion and cell signaling.

In somatic cells, 97% of TEs were characterized by repressive modifications (DNA methylation, H3K9me3, H3K27me3, or H2aK119ub) to control their activity^60^. However, in hPGCs, this percentage significantly decreased to 39%. In female hPGCs, the occupancy of repressive marks on TEs followed the order of DNA methylation + H3K9me3, H3K9me3, DNA methylation, H2aK119ub, and H3K27me3^60^. Some TEs in hPGCs specifically acquired marks such as H3K9me3, H2aK119ub, or H3K27me3, suggesting compensation for the reduction in DNA methylation. The TE groups could be broadly divided into two groups on the basis of the modification: H3K27me3/H2aK119ub and H3K9me3/5mC. Like in mice, H3K27me3/H2aK119ub-marked TEs, such as DNA transposons and L2, appeared to be evolutionarily old, whereas H3K9me3/5mC-marked TEs, such as L1, SVA, or HERVK, appeared to be evolutionarily young (Fig. 2). There was no broad expression difference within hPGCs on the basis of the type of repressive marks^60^.

Transcriptional reactivation of the X chromosome has been observed in Week 4 hPGCs. DNA methylation levels on the X chromosome in female hPGCs appeared to be depleted to a level similar to that in autosomes. When comparing X chromosomes between female and male hPGCs, both exhibited minimal DNA methylation. However, CGI promoter regions showed H3K4me3 signals uniquely in females^66^. This finding suggested the necessity of the active H3K4me3 mark for chromosome reactivation in females. While it might be anticipated that having both X chromosomes active simultaneously in females would result in a doubling of the gene dosage, the actual increase is 1.6-fold^50^. Additionally, active H3K4me3 is more prominent in female hPGCs than in male hPGCs. Repressive marks such as H3K9me3 are also more common in female hPGCs than in male hPGCs, leading to incomplete reactivation of the X chromosome^60,66^.

Compared with other regions, ‘escapee’ genes or TEs show relatively high enrichment of repressive marks such as H3K9me3 and H3K27me3^66^. However, some TEs that escape, particularly those retaining partial DNA methylation, also exhibit high enrichment of the active mark H3K4me3. The hominoid-restricted HERVK and SVA families are characterized by both active (H3K4me3) and repressive (H3K9me3) chromatin marks, revealing a significant correlation between histone methylation and expression. SVA subfamilies show significant differences in expression on the basis of the presence of active H3K4me3 and repressive H3K9me3 marks. Evolutionarily young subfamilies, such as SVA_D and SVA_E, show high H3K4me3 and low H3K9me3, resulting in high expression, whereas older subfamilies like SVA_A and SVA_B prominently show H3K9me3. One subfamily of ‘escapee’ TEs, HERVK/LTR5_Hs, can influence the expression of neighboring genes depending on their activation state. This finding suggests a potential role as an enhancer. However, the developmental significance and genomic regulatory functions of TE activation remain largely unclear.

## In vitro culture system for recapitulating the in vivo development of human PGCs

Here, we focus on recent studies indicating advances in the in vitro reconstitution of human germ cell development. Previous studies have reported the outcomes of studies in mouse models^6,67^.

### Specification of human PGCLCs

The process of inducing hPGCLCs at the specification stage in vitro has been developed to mimic conditions found in vivo. Although BMP signaling can induce hPGCLCs, it is known that a competent cell type capable of initiating PGC fate in response to this signaling is achievable only under specific conditions^20^. Human embryonic stem cells (ESCs) are cultured under 4i conditions (referred to as 4i ESCs), in which chemical inhibitors targeting MEK, GSK3β, p38, and c-Jun N-terminal kinase (JNK) are employed to simulate a perigastrulation epiblast-like state^2,6^. These 4i ESCs, along with transient incipient mesoderm-like cells (iMeLCs) and precursors of the mesoderm (PreME), possess the ability to differentiate into hPGCLCs upon exposure to BMP signaling^68,69^. hPGCLCs can also be differentiated from induced pluripotent stem cells (iPSCs) under 4i conditions, as well as from iPSC-derived iMeLCs^20^. In contrast, ESCs cultured with FGF and transforming growth factor-β (TGFβ) signaling are recognized to exist in a ‘primed’ pluripotent state. They exhibit very low efficiency for hPGCLC differentiation in response to BMP^20^.

The induced hPGCLCs were found to be at the nascent specification stage, and the global DNA methylation level in hPGCLCs was 68%, indicating that genome-wide demethylation had not yet occurred^26^. However, immunofluorescence images revealed that the level of DNA hydroxymethylation in nascent hPGCLCs was greater than that in adjacent somatic cells, indicating the initiation of epigenetic resetting^20^. Extensive research has reported the further differentiation of hPGCLCs into migratory or gonadal stages, which involve epigenetic reprogramming.

### Human PGCLC differentiation beyond specification through recapitulation of the in vivo microenvironment

**Fig. 3:**
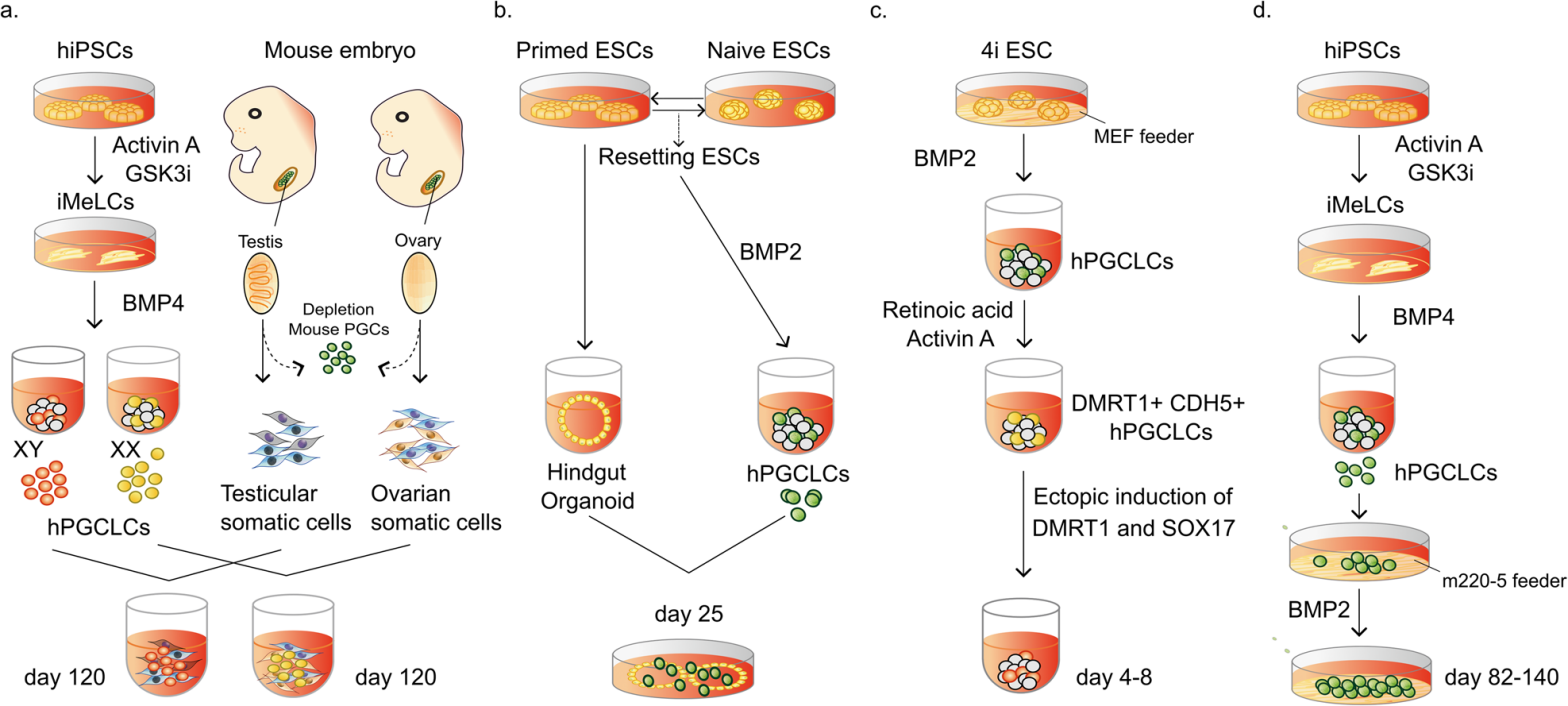
Human in vitro gametogenesis. **a** A methodology for reaggregation culture involving male or female human PGCLCs with mouse embryonic somatic cells was employed. The hPGCLCs from the embryoids were then cocultured for ~4 months, with mouse testicular or ovarian somatic cells differentiating into pro-spermatogonia or oogonia-like cells, respectively. hiPSCs human induced pluripotent stem cells, iMeLCs incipient mesoderm-like cells, GSK3i GSK3 inhibitor. **b** A methodology for coculture with hindgut organoids demonstrated enhanced progression capability in the human germ cell fate. Cells transitioning between primed and naïve pluripotency states can be directed toward hPGCLCs via BMP signaling cues. hPGCLCs cocultured with human hindgut organoids derived from primed ESCs exhibit developmental progression similar to that of in vivo gonadal hPGCs. **c** Ectopic induction of DMRT1 and SOX17 induces the expression of mitotic arrest marker genes, indicating the transition from nascent hPGCLCs to gametogenesis-competent cells. MEF mouse embryonic fibroblast. **d** Prolonged culture with BMP2 promotes the differentiation of hPGCLCs into mitotic pro-spermatogonia or oogonia-like cells.

One attempt to achieve the progression of nascent hPGCLCs has been to aggregate them with mouse embryonic ovarian somatic cells cultured for ~4 months (Fig. 3a). Under these conditions, ~0.5% of hPGCLCs differentiate into oogonia and immediate precursory states for oocytes^70,71^. In vivo, gonadal somatic cells undergo sex-specific differentiation into male- or female-specific cells earlier than PGCs. The signaling and close interactions between somatic cells and PGCs are crucial for their development^72^. Despite the species differences between mice and humans, the xenogeneic reconstituted ovary environment promotes hPGCLCs differentiation.

For coculture with mouse gonadal somatic cells, endogenous mouse PGCs are first removed from E12.5 female gonads via magnetic cell sorting. Human PGCLCs are then mixed with mouse gonadal somatic cells to form aggregates. After ~77 days, the hPGCLCs express the late germ cell genes *DAZL* and *DDX4*. Changes in the gene expression pattern reflect the development of hPGCLCs to a more advanced state. On Day 120, DDX4+ cells exhibit an upregulation of *STRA8*, indicating the initiation of meiosis. Transcriptome analysis suggested that these cells were responsive to signals for meiotic entry in preparation for meiotic recombination^70^. During female germ cell development in vivo, *DAZL* and *DDX4* expression are detected at Week 5, whereas *STRA8* expression is detected in the RA-responsive stage between Weeks 11 and 14^15^. In contrast, this in vitro culture requires more time to reach the oogonia stage.

The global DNA methylation levels decline to ~20% in Day 77 cells and 13% in Day 120 cells. The methylation of the ICRs, a critical indicator of PGC epigenetic reprogramming, is largely but not fully erased. Additionally, the promoters of X-linked genes are moderately demethylated (~20%). In contrast, repeats known as escapees, such as SVA and HERVK, retain higher levels of DNA methylation than other repeats^70^. Overall, if imperfect, the processes are broadly comparable to those of in vivo hPGC differentiation postspecification.

Methods to differentiate hPGCLCs into prespermatogonia through coculture with mouse fetal testicular cells have also been reported (Fig. 3a)^73^. E12.5 mouse fetal testicular cells readily formed reconstituted testes (rTestis), and hPGCLCs could self-assemble to form mini-fetal testicular tissues (xenogeneic reconstituted testis, xrTestis). By Day 120, the xrTestes exhibits scattered DDX4+/DAZL+ cells with significantly reduced expression of *TFAP2C*, *POU5F1*, and *NANOG* (~4% efficiency). Immunofluorescence analysis of the xrTestes on Day 120 revealed a significant reduction in the number of MKI67+ germ cells compared with that of the Day 77 xrTestes, indicating progression into the mitotically arrested state. In these cells, genes involved in transcription factor activity, spermatogenesis, and DNA methylation involved in gamete generation, including those in piRNA pathways known as markers for pro-spermatogonia, are upregulated. In vivo, male PGCs enter the mitotic arrest stage starting at Week 9^15^. This finding suggests that, similar to the female coculture method, a longer period is required for this progression.

After the specification of hPGCs, they migrate to the developing gonad through the hindgut and dorsal mesentery. A study reported that directly differentiating the human hindgut from ESCs and coculturing them with hPGCLCs results in enhanced progression (Fig. 3b)^16^. When hPGCLCs are cocultured with hindgut organoids until Day 25, the expression of genes such as *DAZL* or *DDX4* (3–13% efficiency) increases, and transcriptome analysis revealed that compared with nascent hPGCLCs, hPGCLCs progress toward Week 6 or 8 hPGCs in vivo. Compared with their neighboring somatic cells, hPGCLCs cocultured with human hindgut organoids exhibit a reduction in 5mC and H3K9me2, as observed through immunofluorescence analysis.

### Human PGCLC differentiation beyond specification through the modulation of signaling pathways and transcription factors

Although coculture with mouse embryonic ovarian or testicular cells, or hindgut organoids, can promote differentiation beyond PGC specification, the precise molecular mechanisms remain to be fully elucidated. The regulatory factors and mechanisms involved in PGC migration and the transition to oogonia or pro-spermatogonia must also be explored fully. However, recent research has revealed that the DMRT1 transcription factor plays a role in differentiating hPGCs postspecification^17^. In vivo, *DMRT1* expression begins during the migration stage of hPGCs and increases significantly at the gonadal stage. When BMP is used to induce nascent hPGCLCs for specification, followed by treatment with Activin A and retinoic acid signaling, there is an increase in the expression of genes associated with the migratory stage, such as *DMRT1* and *CDH5*^17^. Furthermore, ectopic induction of DMRT1 and SOX17 in these cells leads to the upregulation of genes specifically expressed during the mitotic arrest stage in male hPGCs, including late germ cell genes such as *DAZL* (Fig. 3c). Conversely, it suppresses the pluripotency genes *POU5F1* and *NANOG*, as observed in male mitotic arrest and female RA-responsive hPGCs. The induction of DMRT1 and SOX17 in the female line results in DAZL+ hPGCLCs, which also exhibit a downregulation of pluripotency genes. These findings suggest a common role for DMRT1 in early germline commitment in both males and females. In female hPGCs, *DMRT1* expression decreases with meiotic entry following the RA-responsive stage^17^.

Global epigenetic changes were also observed, with DNA hydroxymethylation levels increasing by ~10% in DAZL+ hPGCLCs. This increase occurs not only at the genomic level but is particularly pronounced for genes expressed specifically in mitotic arrest male hPGCs. This phenomenon is associated with DMRT1 targeting, since DNA hydroxymethylation is more pronounced at DMRT1 binding sites, accompanied by decreased DNA methylation^17^. However, localized changes are evident, while the global DNA methylation levels do not significantly decrease, as observed in in vivo mitotic arrest hPGCs. DMRT1-induced mitotic arrest may prevent replication-coupled loss of DNA methylation. A gradual increase in DMRT1 dosage may permit extensive DNA demethylation and appropriate developmental progression of germ cells. An interesting finding concerning the regulation by DMRT1 is its effect on TEs. TEs such as SVA and LTR12C, which are strongly expressed in hPGCs at the gonadal PGC stage, show increased expression upon DMRT1 induction. Conversely, TEs such as LTR5HS and HERVK-int present decreased expression, mirroring the expression patterns observed during in vivo hPGC development^17^.

Recent studies have reported methods for differentiating mitotic pro-spermatogonia or oogonia accompanied by global DNA demethylation (Fig. 3d)^33^. After hPGCLC specification, continuous stimulation with the WNT signaling inhibitor IWR1 and BMP allows hPGCLCs to propagate and progress to the gonadal stage. According to the results of this study, BMP signaling is crucial for the further differentiation of hPGCs, as evidenced by the expression levels of genes such as *DAZL* and *DDX4*. Increasing the concentration of BMP2 results in a greater proportion of DAZL+ cells (Day 82: ~15%, Day 140: ~80%); without BMP2 under the same conditions, hPGCLCs undergo dedifferentiation. For female hPGCLCs, single-cell RNA-seq analysis of cultured cells revealed a gradual differentiation process from the mitotic stage to the meiotic stage (zygotene/pachytene/diplotene, or ZPD), comparable to in vivo hPGC differentiation, although the process occurs over an extended period that is not observed in vivo. Notably, this process was accompanied by DNA methylome reprogramming, with DNA methylation levels declining to ~10% upon BMP-driven hPGCLC differentiation^33^. This demethylation is observed in genes associated with the meiotic cell cycle and ICRs, with nearly all imprints erased except for *PEG3*, *IGF2R*, and *ZFAT*. However, methylation in escapee regions evades demethylation, and X chromosome demethylation is incomplete, with ~24% remaining on Day 127^33^.

## Concluding remarks

The development of germ cells, which generate the totipotent state at fertilization, begins around the 2nd week with the specification of PGCs and continues through puberty. A relatively small number of cells acquire the PGC fate, which diverges from that of somatic cells. PGC specification is linked with initiating global epigenetic resetting, including global DNA methylation erasure, which starts as PGCs migrate after their specification from the epiblast. This process continues as the cells reach the developing gonads, sequentially acquiring gamete-specific epigenomic status. Locus-specific histone modifications accompany DNA demethylation and ensure an orderly program for germ cell development. Repressive histone modifications generally decrease in PGCs, but to complement DNA demethylation, developmental genes or specific groups of TEs are markedly distinct in PGCs compared with those in somatic cells, helping to maintain a repressed state. Furthermore, there are differences in histone modification between males and females, indicating sex-specific regulation of germ cells.

Technological advances have recently elucidated many aspects of PGC development. However, technical and ethical challenges have left significant gaps, particularly in the study of hPGCs. hPGC development requires in vitro culture systems because species-specific developmental processes cannot be adequately modeled in mice. Various approaches have been employed, including coculture techniques that mimic the migratory path and the gonadal environment and the modulation of specific signaling factors and transcription factors to induce differentiation of hPGCLCs postspecification. By comparing the transcriptome and epigenetic status of cells differentiated in vitro with those observed in hPGCs in vivo, researchers have assessed the extent of differentiation and successfully replicated global epigenetic changes unique to hPGCs. These studies are fundamental for exploring and understanding the principles of germ cell development for reproduction, which is one of the most crucial biological processes. Moreover, they contribute to advancements in reproductive medicine and potential treatments for infertility. Understanding the intricate process of hPGC development can aid in the development of therapies for various reproductive disorders and improve assisted reproductive technologies.

## References

[1] Seydoux, G. & Braun, R. E. Pathway to totipotency: lessons from germ cells. *Cell* **127**, 891–904 (2006). 17129777 10.1016/j.cell.2006.11.016

[2] Tang, W. W., Kobayashi, T., Irie, N., Dietmann, S. & Surani, M. A. Specification and epigenetic programming of the human germ line. *Nat. Rev. Genet.* **17**, 585–600 (2016). 27573372 10.1038/nrg.2016.88

[3] Czukiewska, S. M. & Chuva de Sousa Lopes, S. M. Fetal germ cell development in humans, a link with infertility. *Semin. Cell Dev. Biol.* **131**, 58–65 (2022). 35431137 10.1016/j.semcdb.2022.03.035

[4] Mamsen, L. S., Brochner, C. B., Byskov, A. G. & Mollgard, K. The migration and loss of human primordial germ stem cells from the hind gut epithelium towards the gonadal ridge. *Int J. Dev. Biol.* **56**, 771–778 (2012). 23417399 10.1387/ijdb.120202lm

[5] Kobayashi, T. et al. Tracing the emergence of primordial germ cells from bilaminar disc rabbit embryos and pluripotent stem cells. *Cell Rep.* **37**, 109812 (2021). 34644585 10.1016/j.celrep.2021.109812

[6] Saitou, M. & Hayashi, K. Mammalian in vitro gametogenesis. *Science* **374**, eaaz6830 (2021). 34591639 10.1126/science.aaz6830

[7] Zhu, Q. et al. Specification and epigenomic resetting of the pig germline exhibit conservation with the human lineage. *Cell Rep.* **34**, 108735 (2021). 33567277 10.1016/j.celrep.2021.108735 PMC7873836

[8] Messerschmidt, D. M., Knowles, B. B. & Solter, D. DNA methylation dynamics during epigenetic reprogramming in the germline and preimplantation embryos. *Genes Dev.* **28**, 812–828 (2014). 24736841 10.1101/gad.234294.113 PMC4003274

[9] Tang, W. W. et al. A unique gene regulatory network resets the human germline epigenome for development. *Cell* **161**, 1453–1467 (2015). 26046444 10.1016/j.cell.2015.04.053 PMC4459712

[10] Ohinata, Y. et al. A signaling principle for the specification of the germ cell lineage in mice. *Cell* **137**, 571–584 (2009). 19410550 10.1016/j.cell.2009.03.014

[11] Molyneaux, K. A., Stallock, J., Schaible, K. & Wylie, C. Time-lapse analysis of living mouse germ cell migration. *Dev. Biol.* **240**, 488–498 (2001). 11784078 10.1006/dbio.2001.0436

[12] Tam, P. P. & Snow, M. H. Proliferation and migration of primordial germ cells during compensatory growth in mouse embryos. *J. Embryol. Exp. Morphol.* **64**, 133–147 (1981). 7310300

[13] Mayere, C. et al. Single-cell transcriptomics reveal temporal dynamics of critical regulators of germ cell fate during mouse sex determination. *FASEB J.* **35**, e21452 (2021). 33749946 10.1096/fj.202002420R

[14] Zhao, J. et al. Cell-fate transition and determination analysis of mouse male germ cells throughout development. *Nat. Commun.* **12**, 6839 (2021). 34824237 10.1038/s41467-021-27172-0 PMC8617176

[15] Li, L. et al. Single-cell RNA-seq analysis maps development of human germline cells and gonadal niche interactions. *Cell Stem Cell* **20**, 891–892 (2017). 28575695 10.1016/j.stem.2017.05.009

[16] Alves-Lopes, J. P. et al. Specification of human germ cell fate with enhanced progression capability supported by hindgut organoids. *Cell Rep.* **42**, 111907 (2023). 36640324 10.1016/j.celrep.2022.111907 PMC7618081

[17] Irie, N. et al. DMRT1 regulates human germline commitment. *Nat. Cell Biol.* **25**, 1439–1452 (2023). 37709822 10.1038/s41556-023-01224-7 PMC10567552

[18] Ohinata, Y. et al. Blimp1 is a critical determinant of the germ cell lineage in mice. *Nature* **436**, 207–213 (2005). 15937476 10.1038/nature03813

[19] Weber, S. et al. Critical function of AP-2gamma/TCFAP2C in mouse embryonic germ cell maintenance. *Biol. Reprod.* **82**, 214–223 (2010). 19776388 10.1095/biolreprod.109.078717

[20] Irie, N. et al. SOX17 is a critical specifier of human primordial germ cell fate. *Cell* **160**, 253–268 (2015). 25543152 10.1016/j.cell.2014.12.013 PMC4310934

[21] Tang, W. W. C. et al. Sequential enhancer state remodelling defines human germline competence and specification. *Nat. Cell Biol.* **24**, 448–460 (2022). 35411086 10.1038/s41556-022-00878-z PMC7612729

[22] Kojima, Y. et al. GATA transcription factors, SOX17 and TFAP2C, drive the human germ-cell specification program. *Life Sci. Alliance* **4**, 10.26508/lsa.202000974 (2021). 10.26508/lsa.202000974 PMC7918644 33608411

[23] Campolo, F. et al. Essential role of Sox2 for the establishment and maintenance of the germ cell line. *Stem Cells* **31**, 1408–1421 (2013). 23553930 10.1002/stem.1392

[24] Tam, P. P. & Behringer, R. R. Mouse gastrulation: the formation of a mammalian body plan. *Mech. Dev.* **68**, 3–25 (1997). 9431800 10.1016/s0925-4773(97)00123-8

[25] Rossant, J. & Tam, P. P. L. Early human embryonic development: blastocyst formation to gastrulation. *Dev. Cell* **57**, 152–165 (2022). 35077679 10.1016/j.devcel.2021.12.022

[26] von Meyenn, F. et al. Comparative principles of DNA methylation reprogramming during human and mouse in vitro primordial germ cell specification. *Dev. Cell* **39**, 104–115 (2016). 27728778 10.1016/j.devcel.2016.09.015 PMC5064768

[27] Lee, S. M. Detecting DNA hydroxymethylation: exploring its role in genome regulation. *BMB Rep.* **57**, 135–142 (2024). 38449301 10.5483/BMBRep.2023-0250 PMC10979348

[28] Li, E. & Zhang, Y. DNA methylation in mammals. *Cold Spring Harb. Perspect. Biol.* **6**, a019133 (2014). 24789823 10.1101/cshperspect.a019133 PMC3996472

[29] Tahiliani, M. et al. Conversion of 5-methylcytosine to 5-hydroxymethylcytosine in mammalian DNA by MLL partner TET1. *Science* **324**, 930–935 (2009). 19372391 10.1126/science.1170116 PMC2715015

[30] He, Y. F. et al. Tet-mediated formation of 5-carboxylcytosine and its excision by TDG in mammalian DNA. *Science* **333**, 1303–1307 (2011). 21817016 10.1126/science.1210944 PMC3462231

[31] Guo, F. et al. Active and passive demethylation of male and female pronuclear DNA in the mammalian zygote. *Cell Stem Cell* **15**, 447–459 (2014). 25220291 10.1016/j.stem.2014.08.003

[32] Hill, P. W. S. et al. Epigenetic reprogramming enables the transition from primordial germ cell to gonocyte. *Nature* **555**, 392–396 (2018). 29513657 10.1038/nature25964 PMC5856367

[33] Murase, Y. et al. In vitro reconstitution of epigenetic reprogramming in the human germ line. *Nature* **631**, 170–178 (2024). 38768632 10.1038/s41586-024-07526-6 PMC11222161

[34] Liu, X. L. et al. UHRF1 targets DNMT1 for DNA methylation through cooperative binding of hemi-methylated DNA and methylated H3K9. *Nat. Commun.* **4**, 10.1038/ncomms2562 (2013). 10.1038/ncomms2562 23463006

[35] Guo, H. et al. The DNA methylation landscape of human early embryos. *Nature* **511**, 606–610 (2014). 25079557 10.1038/nature13544

[36] Frommer, M. et al. A genomic sequencing protocol that yields a positive display of 5-methylcytosine residues in individual DNA strands. *Proc. Natl. Acad. Sci. USA* **89**, 1827–1831 (1992). 1542678 10.1073/pnas.89.5.1827 PMC48546

[37] Yan, R. et al. Dynamics of DNA hydroxymethylation and methylation during mouse embryonic and germline development. *Nat. Genet.* **55**, 130–143 (2023). 36539615 10.1038/s41588-022-01258-x

[38] Guo, F. et al. Single-cell multi-omics sequencing of mouse early embryos and embryonic stem cells. *Cell Res.* **27**, 967–988 (2017). 28621329 10.1038/cr.2017.82 PMC5539349

[39] Wang, L. et al. Programming and inheritance of parental DNA methylomes in mammals. *Cell* **157**, 979–991 (2014). 24813617 10.1016/j.cell.2014.04.017 PMC4096154

[40] Ivanova, E. et al. DNA methylation changes during preimplantation development reveal inter-species differences and reprogramming events at imprinted genes. *Clin. Epigenetics* **12**, 10.1186/s13148-020-00857-x (2020). 10.1186/s13148-020-00857-x PMC7216732 32393379

[41] Seisenberger, S. et al. The dynamics of genome-wide DNA methylation reprogramming in mouse primordial germ cells. *Mol. Cell* **48**, 849–862 (2012). 23219530 10.1016/j.molcel.2012.11.001 PMC3533687

[42] Auclair, G., Guibert, S., Bender, A. & Weber, M. Ontogeny of CpG island methylation and specificity of DNMT3 methyltransferases during embryonic development in the mouse. *Genome Biol.* **15**, 545 (2014). 25476147 10.1186/s13059-014-0545-5 PMC4295324

[43] Guo, H. et al. DNA methylation and chromatin accessibility profiling of mouse and human fetal germ cells. *Cell Res.* **27**, 165–183 (2017). 27824029 10.1038/cr.2016.128 PMC5339845

[44] Kobayashi, H. et al. High-resolution DNA methylome analysis of primordial germ cells identifies gender-specific reprogramming in mice. *Genome Res.* **23**, 616–627 (2013). 23410886 10.1101/gr.148023.112 PMC3613579

[45] Zhu, P. et al. Single-cell DNA methylome sequencing of human preimplantation embryos. *Nat. Genet.* **50**, 12–19 (2018). 29255258 10.1038/s41588-017-0007-6

[46] Li, C. et al. DNA methylation reprogramming of functional elements during mammalian embryonic development. *Cell Discov.* **4**, 41 (2018). 30109120 10.1038/s41421-018-0039-9 PMC6079081

[47] Hernandez Mora, J. R. et al. Single-cell multi-omic analysis profiles defective genome activation and epigenetic reprogramming associated with human pre-implantation embryo arrest. *Cell Rep.* **42**, 112100 (2023). 36763500 10.1016/j.celrep.2023.112100

[48] Zhou, F. et al. Reconstituting the transcriptome and DNA methylome landscapes of human implantation. *Nature* **572**, 660–664 (2019). 31435013 10.1038/s41586-019-1500-0

[49] Li, L. et al. Dissecting the epigenomic dynamics of human fetal germ cell development at single-cell resolution. *Cell Res.* **31**, 463–477 (2021). 32884136 10.1038/s41422-020-00401-9 PMC8115345

[50] Guo, F. et al. The transcriptome and DNA methylome landscapes of human primordial germ cells. *Cell* **161**, 1437–1452 (2015). 26046443 10.1016/j.cell.2015.05.015

[51] Hackett, J. A. et al. Germline DNA demethylation dynamics and imprint erasure through 5-hydroxymethylcytosine. *Science* **339**, 448–452 (2013). 23223451 10.1126/science.1229277 PMC3847602

[52] Kidwell, M. G. & Lisch, D. Transposable elements as sources of variation in animals and plants. *Proc. Natl. Acad. Sci. USA* **94**, 7704–7711 (1997). 9223252 10.1073/pnas.94.15.7704 PMC33680

[53] Schumann, G. G. et al. The impact of transposable element activity on therapeutically relevant human stem cells. *Mob. DNA* **10**, 9 (2019). 30899334 10.1186/s13100-019-0151-x PMC6408843

[54] Rebollo, R. et al. Inter-strain epigenomic profiling reveals a candidate IAP master copy in C3H mice. *Viruses* **12**, 10.3390/v12070783 (2020). 10.3390/v12070783 PMC7411935 32708087

[55] Jones, P. A. Functions of DNA methylation: islands, start sites, gene bodies and beyond. *Nat. Rev. Genet.* **13**, 484–492 (2012). 22641018 10.1038/nrg3230

[56] Lee, S. M., Choi, W. Y., Lee, J. & Kim, Y. J. The regulatory mechanisms of intragenic DNA methylation. *Epigenomics* **7**, 527–531 (2015). 26111026 10.2217/epi.15.38

[57] Zhou, W., Liang, G., Molloy, P. L. & Jones, P. A. DNA methylation enables transposable element-driven genome expansion. *Proc. Natl. Acad. Sci. USA* **117**, 19359–19366 (2020). 32719115 10.1073/pnas.1921719117 PMC7431005

[58] Huang, T. C. et al. Sex-specific chromatin remodelling safeguards transcription in germ cells. *Nature* **600**, 737–742 (2021). 34880491 10.1038/s41586-021-04208-5

[59] Ng, J. H. et al. In vivo epigenomic profiling of germ cells reveals germ cell molecular signatures. *Dev. Cell* **24**, 324–333 (2013). 23352811 10.1016/j.devcel.2012.12.011

[60] Gruhn, W. H. et al. Epigenetic resetting in the human germ line entails histone modification remodeling. *Sci. Adv.* **9**, eade1257 (2023). 36652508 10.1126/sciadv.ade1257 PMC9848478

[61] Vallender, E. J., Mekel-Bobrov, N. & Lahn, B. T. Genetic basis of human brain evolution. *Trends Neurosci.* **31**, 637–644 (2008). 18848363 10.1016/j.tins.2008.08.010 PMC2715140

[62] Nozawa, M., Kawahara, Y. & Nei, M. Genomic drift and copy number variation of sensory receptor genes in humans. *Proc. Natl. Acad. Sci. USA* **104**, 20421–20426 (2007). 18077390 10.1073/pnas.0709956104 PMC2154446

[63] Feber, A. et al. Using high-density DNA methylation arrays to profile copy number alterations. *Genome Biol.* **15**, R30 (2014). 24490765 10.1186/gb-2014-15-2-r30 PMC4054098

[64] Shi, X. et al. Association of CNVs with methylation variation. *NPJ Genom. Med.* **5**, 41 (2020). 33062306 10.1038/s41525-020-00145-w PMC7519119

[65] Shi, T. H., Sugishita, H. & Gotoh, Y. Crosstalk within and beyond the Polycomb repressive system. *J. Cell Biol.* **223**, 10.1083/jcb.202311021 (2024). 10.1083/jcb.202311021 PMC10955045 38506728

[66] Gao, R. et al. Resetting histone modifications during human prenatal germline development. *Cell Discov.* **9**, 14 (2023). 36737434 10.1038/s41421-023-00519-1 PMC9898496

[67] Ishikura, Y. et al. In vitro reconstitution of the whole male germ-cell development from mouse pluripotent stem cells. *Cell Stem Cell* **28**, 2167–2179.e2169 (2021). 34496297 10.1016/j.stem.2021.08.005

[68] Sasaki, K. et al. Robust in vitro induction of human germ cell fate from pluripotent stem cells. *Cell Stem Cell* **17**, 178–194 (2015). 26189426 10.1016/j.stem.2015.06.014

[69] Kobayashi, T. et al. Principles of early human development and germ cell program from conserved model systems. *Nature* **546**, 416–420 (2017). 28607482 10.1038/nature22812 PMC5473469

[70] Yamashiro, C. et al. Generation of human oogonia from induced pluripotent stem cells in vitro. *Science* **362**, 356–360 (2018). 30237246 10.1126/science.aat1674

[71] Yamashiro, C., Sasaki, K., Yokobayashi, S., Kojima, Y. & Saitou, M. Generation of human oogonia from induced pluripotent stem cells in culture. *Nat. Protoc.* **15**, 1560–1583 (2020). 32231324 10.1038/s41596-020-0297-5

[72] Overeem, A. W., Chang, Y. W., Spruit, J., Roelse, C. M. & Lopes, S. M. C. D. Ligand-receptor interactions elucidate sex-specific pathways in the trajectory from primordial germ cells to gonia during human development. *Front. Cell Dev. Biol.* **9**, 10.3389/fcell.2021.661243 (2021). 10.3389/fcell.2021.661243 PMC8253161 34222234

[73] Hwang, Y. S. et al. Reconstitution of prospermatogonial specification in vitro from human induced pluripotent stem cells. *Nat. Commun.* **11**, 5656 (2020). 33168808 10.1038/s41467-020-19350-3 PMC7653920

